# Inhibition of HLA-DM Mediated MHC Class II Peptide Loading by HLA-DO Promotes Self Tolerance

**DOI:** 10.3389/fimmu.2013.00465

**Published:** 2013-12-17

**Authors:** Lisa K. Denzin

**Affiliations:** ^1^Department of Pediatrics, Robert Wood Johnson Medical School, Child Health Institute of New Jersey, Rutgers, The State University of New Jersey, New Brunswick, NJ, USA

**Keywords:** antigen presentation, MHC class II, HLA-DM, HLA-DO, autoimmunity, germinal center reaction, non-obese diabetic, diabetes

## Abstract

Major histocompatibility class II (MHCII) molecules are loaded with peptides derived from foreign and self-proteins within the endosomes and lysosomes of antigen presenting cells (APCs). This process is mediated by interaction of MHCII with the conserved, non-polymorphic MHCII like molecule HLA-DM (DM). DM activity is directly opposed by HLA-DO (DO), another conserved, non-polymorphic MHCII like molecule. DO is an MHCII substrate mimic. Binding of DO to DM prevents MHCII from binding to DM, thereby inhibiting peptide loading. Inhibition of DM function enables low stability MHC complexes to survive and populate the surface of APCs. As a consequence, DO promotes the display of a broader pool of low abundance self-peptides. Broadening the peptide repertoire theoretically reduces the likelihood of inadvertently acquiring a density of self-ligands that is sufficient to activate self-reactive T cells. One function of DO, therefore, is to promote T cell tolerance by shaping the visible image of self. Recent data also shows that DO influences the adaptive immune response by controlling B cell entry into the germinal center reaction. This review explores the data supporting these concepts.

## Introduction

The detection of major histocompatibility class II (MHCII) molecules displaying pathogen-derived peptides on antigen presenting cells (APCs) by CD4 T cells is an essential step in the initiation of adaptive immune responses. Counterintuitively, the majority of surface expressed MHCII peptide (pMHCII) complexes do not display pathogen-derived peptides. Rather, they are filled with self-peptides. CD4 T cells are immunologically tolerant of self-pMHCII complexes since inappropriate T cell activation contributes to the development of autoimmune disease (1). The presentation of self-pMHCII complexes at appropriate times plays an important role in tolerance by inducing the death of self-reactive thymocytes or by rendering mature CD4 T cells functionally non-responsive. Thus, tight control of MHCII peptide loading is important for not only CD4 T cell activation to foreign antigens, but also for establishing and maintaining CD4 T cell tolerance. The binding of specific peptides to MHC is largely controlled by the structural constraints inherent to allelic variations in MHCII. However, MHCII peptide loading is impacted by other mechanisms including antigen delivery into endosomal compartments, proteolysis and by direct modulation of MHCII peptide loading by the accessory molecules HLA-DM (DM) and HLA-DO (DO) (2).

The basic biosynthetic and cellular pathways by which MHCII molecules acquire their peptide cargo are reviewed elsewhere (3, 4). Briefly, MHCII αβ heterodimers associate with the non-polymorphic invariant chain (Ii) during assembly in the endoplasmic reticulum. Ii occupies the peptide binding groove of the MHCII preventing unfolded proteins and peptides from prematurely binding to MHCII in the ER. Ii also targets MHCII-Ii complexes to late endosomal and lysosomal compartments where Ii is degraded by resident proteases. Ii is not degraded to completion, instead small fragments of Ii, termed class II-associated Ii peptides or CLIP remain bound in the MHCII peptide groove (5, 6). CLIP must be removed from the groove prior to binding of peptides derived from self and foreign antigens. This process is mediated by interaction of the non-classical MHCII like molecule, DM (H2-M in mice) with MHCII-CLIP complexes (7). DM also stabilizes an empty and peptide receptive form of MHCII and edits the repertoire of MHCII-bound peptides ensuring that exceptionally stable pMHCII complexes are presented at the cell surface (7, 8). Although it is recognized that DM is essential for MHCII antigen presentation, the molecular mechanisms by which DM mediates MHCII peptide loading remain unclear. Previous studies combined with recent biochemical and crystallographic studies, however, have shown that DM forms stable complexes with empty MHCII (i.e., a short-lived transition state) and that this interaction is disrupted by high-affinity peptide binding to MHCII. Thus, when mixtures of low and high-affinity peptides are present, as would be the case *in vivo* in endosomal and lysosomal compartments, DM favors the loading and subsequent cell surface presentation of high stability pMHCII complexes (8–10).

DM mediated peptide loading of MHCII molecules is controlled in DCs, B cells, and medullary thymic epithelial cells by the interaction of DM with another class II-like molecule, DO (H2-O in mice) (11). Although DO and DM are both non-classical MHCII proteins, DO is structurally more closely related to classical MHCII proteins suggesting that DO might interact with DM in a similar manner to that of MHCII. DM and MHCII first interact in endosomal compartments. In contrast, DM-DO association is initiated in the ER and the complex is maintained during and after transport to endosomal compartments (12). Additionally, DM-MHCII interactions are weak, whereas DM-DO complexes are exceptionally stable. The tight association of DM with DO suggested that DO might alter DM function. Initial biochemical studies showed that DM-DO complexes were completely inactive in terms of their ability to catalyze MHCII peptide loading *in vitro*, supporting that DO was an inhibitor of DM function (13, 14). Human cell line-based transfection studies supported this idea and showed that cells expressing DO have high levels of MHCII-CLIP where as DO-negative cells have comparably lower levels (15). Additionally, DO/H2-O is down regulated upon DC and B cell activation, freeing DM from DO inhibition, presumably resulting in optimal MHCII peptide loading upon pathogen encounter *in vivo* (16–21). Collectively, these studies supported that DO inhibited DM.

The impact of DO on DM function became less clear, however, when the consequence of DO expression on the presentation of individual antigenic peptides was measured. These studies, using pMHCII specific Abs, TCR transgenic T cells, primary T cell lines, or T cell hybridomas to measure presentation, showed that DO/H2-O expression unpredictably affected the presentation of individual antigenic peptides (15). DO expression inhibits or enhances presentation of some peptides, while the presentation of other peptides is not affected. These results lead to the idea that DO was not simply an inhibitor of DM but instead somehow it both positively and negatively attenuated DM function.

**Figure 1:**
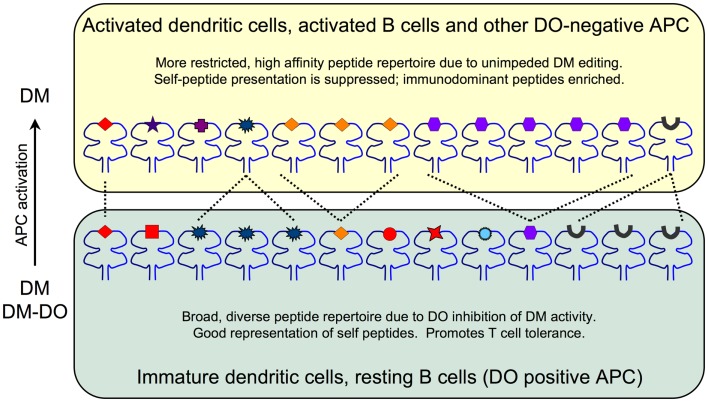
**The impact of DO inhibition of DM on the MHCII-bound peptide repertoire**. Immature DCs and resting B cells (bottom) express high levels of DO and thus have pools of active endosomal DM as well as inactive DM-DO complexes that shape the overall peptide repertoire. The net result is a broad, diverse set of lower stability peptides presented on MHCII. Self-peptides (red) including CLIP (black peptide) are well represented. Activated DCs and B cells and other DO-negative APC (top) are enriched for DM due to DO downregulation and thus display a more focused and high stability peptide repertoire. Self-peptide presentation including CLIP is reduced at the expense of high stability peptide presentation promoted by fully active DM editing.

Recent crystallographic, biochemical and mutagenesis studies, however, demonstrated that DO is undeniably an irreversible inhibitor of DM, in agreement with initial biochemical studies (22, 23). These studies showed that DO interacts with DM in a manner that is nearly identical to MHCII. Thus, DO inhibits DM by acting as a MHCII substrate mimic. The effect that DO has on the pMHC repertoire is due to DO sequestering DM and preventing it from functionally acting on MHCII to promote and/or edit peptide loading. These data support a model in which the net result of DO expression is an overall changed peptide repertoire with overall increased peptide diversity displayed at the cell surface in the context of MHCII (Figure 1). In resting APCs, high levels of DO inhibit DM, thereby promoting the presentation of low stability peptides; self-derived peptides that normally would be edited from MHCII by free DM (i.e., not associated with DO). The likely consequence is that resting APCs present a more complex/diverse peptide repertoire. In contrast, when DO expression is low or absent, such as in activated APCs, MHCII-bound peptides are enriched for those with higher stability, such as many immunodominant peptides. Importantly, DM activity is never completely extinguished by DO. In primary resting APCs (high DO), DM is always expressed in excess of DO with estimates indicating that ~30–50% of DM is free (16, 24). Thus, the final pMHCII repertoire is controlled by the ratio of free, active DM relative to the pool of inactive DM-DO. Although many studies show that DO expression alters peptide loading of MHCII, direct evidence showing that DO affects the overall kinetic stability of the cell surface MHCII-bound peptide repertoire is lacking. Nevertheless, as discussed below, even potentially small changes in pMHCII presentation induced by DO can have a profound impact on immune responses and modulate autoimmunity.

## H2-O Inhibits *In vivo* Immune Responses

Essential functions for DM in the MHCII antigen presentation pathway have been established, however a role for DO has remained enigmatic (2). While it is apparent that DO is a MHCII mimic that binds to and inhibits DM, the biological consequences of DO inhibition of DM are less obvious. The generation and characterization of H2-O deficient mice have, for the most part, been disappointing in terms of providing decisive information about H2-O function *in vivo*. Nevertheless, limited peptide sequencing studies and antigen presentation assays show that H2-O expression results in an altered peptide repertoire (2, 25, 26).

The effect of H2-O expression on the presentation of individual epitopes is typically quite small (<2-fold) and variable (15). The small effect is likely due to the presence of both active H2-M and inactive H2-M/H2-O complexes; H2-O does not completely extinguish H2-M activity. Most proteins, especially large proteins, have multiple epitopes that bind MHCII and are presented. The impact of H2-O on the presentation of individual peptides might be unimpressive, yet the sum of changes for multiple epitopes from a single antigen might significantly impact the immune response. To test this idea, we set up a sensitive *in vivo* system to measure antigen presentation (27).

Entrance of B cells into the germinal center response is a competitive process that is dependent upon help from CD4 T follicular helper cells (28). B cells expressing higher densities of specific pMHCII complexes more effectively receive T cell help and differentiate into germinal center B cells. A competition between antigen specific B cells from wild type and H2-O deficient mice was established by simultaneous transfer into wild type recipient mice. After immunization, the ability of the transferred B cells to present pMHCII, interact with T follicular helper cells and become germinal center B cells was measured. For both antigens tested in this system, H2-O deficient antigen specific B cells won the competition (~2- to 5-fold) (27). When presentation by the antigen specific B cells was directly measured *ex vivo* with primary, polyclonal CD4 T cells, H2-O deficient antigen specific B cells presented slightly better (<2-fold). Nevertheless, the net effect of the small change in pMHCII levels enabled the H2-O deficient B cells to preferentially obtain T cell help and become germinal center B cells. Thus, H2-O expression established a higher threshold of presentation for B cells to join the germinal center reaction. Importantly, downregulation of H2-O upon GC entry removes this barrier to presentation (16, 17). Germinal center B cells undergo somatic hypermutation combined with rapid cellular proliferation, which is inherently dangerous. Thus, inhibition of antigen presentation by H2-O may limit the ability of B cells to gain access to the germinal center in the absence of a significant immunological challenge. In turn this helps to prevent the negative consequences of the germinal center reaction such as lymphoma and leukemia.

Recent studies also examined the impact of H2-O deficiency on B cell immune responses (29). These studies showed that H2-O deficient mice had slightly reduced IgG responses to two model antigens early in the immune response (7 days) but not after. *Ex vivo* antigen presentation assays showed that presentation of the two antigens by wild type splenocytes was slightly better than by H2-O deficient cells. As in our studies, the effect of H2-O on presentation was subtle (≤2-fold) and may explain the initial decrease in humoral responses in H2-O deficient mice. It is not completely clear why these experiments support a positive role for H2-O in generating humoral responses whereas our own studies supported an inhibitory role. Perhaps it was because the two experimental systems assayed different aspects of B cell biology. In our transfer system, antigen specific (i.e., transgenic) wild type and H2-O deficient B cells were transferred together into a wild type recipient, removing potential differences in CD4 T cell repertories or B cell populations (27). In the other study, H2-O deficient and wild type mice were immunized directly (29). The delayed humoral response in the H2-O deficient mice in these experiments might actually be due to reported changes in the CD4 T cell repertoire. The impact of H2-O *in vivo* during immune responses is complex, thus, it will be important to continue to use sensitive immunological systems to completely understand the function of this molecule.

## Mouse Models to Study DO/H2-O Function

Primary human DCs and B cells show a direct correlation between MHCII-CLIP levels and DO expression (15). MHCII-CLIP and DO levels are high in naïve B cells, whereas activated germinal center B cells have low levels of MHC-CLIP and DO, despite maintaining high levels of DM (16, 17). Similar results are observed for resting and activated human DCs (19). Thus, MHCII-CLIP levels are a good surrogate readout for DO levels in human APCs. MHCII-CLIP levels, however, do not correlate with H2-O levels in mouse APCs, despite similar downregulation of H2-O upon APC activation (18, 20, 21, 30). Additionally, MHCII-CLIP levels are only marginally increased on the surface of mouse APCs from H2-O deficient mice (29). Why MHCII-CLIP levels correlate with DO expression in human cells but not mouse cells is unknown. One possibility is that mouse B cells have a pool of free H2-O (20, 30) whereas in human cells, DO is always found in a complex with DM. This implies that the H2-M/H2-O complexes may be less stable than the DM/DO complexes. Alternatively, there might be functional differences between the mouse and human proteins. This possibility is supported by data from transgenic mice we generated that expressed human DO in mouse DCs using the CD11c promoter (CD1c-DO).

In these mice, similar to wild type APC, ~70% of H2-M was complexed with DO in CD11c-DO transgenic mice and DO levels were downregulated upon DC activation, showing that DO functionally interacted with H2-M (21, 31). CD11c-DO DCs displayed an altered set of MHCII-bound peptides compared to DCs from non-transgenic mice when analyzed with monoclonal antibodies (31). As DO expression increased, MHCII-CLIP also increased while pMHCII levels decreased. This was identical to what was observed for human APC. The increased inhibitory activity of DO compared to H2-O might be due to higher affinity binding of DO to H2-M. However the DO inhibitory activity was removed by downregulation of DO in activated DCs, resulting in normal humoral responses upon immunization with model antigens (31, 32). Furthermore, measuring antigen presentation by CD11c-DO DCs to model antigens showed minimal differences (31). Thus, DO expression had a profound impact on the display of self-pMHCII at the cell surface but did not compromise immunity.

## DO/H2-O and Autoimmunity

The biologically relevant question in terms of DO/H2-O function is why is an inhibitor of DM mediated peptide loading necessary for proper function of the immune system? As an inhibitor of DM, the net effect of DO is not only to dampen overall presentation, but also to broaden the peptide repertoire by allowing lower stability peptides to remain bound to MHCII. This idea is important when considering that the majority of peptides presented in non-activated APC are self-peptides. Thus, DO expression ensures that a broad, low-level representation of self is presented. Full T cell activation requires the specific engagement of multiple pMHC complexes. Therefore, increased peptide diversity leads to a decreased likelihood of activating self-reactive CD4 T cells. Thus, DO/H2-O may help mediate tolerance and prevent the development of autoimmune disease.

The presentation of islet-derived self-peptides by MHCII molecules on DCs is essential for the initiation of the T cell mediated autoimmune diseases, such as type 1 diabetes (T1D) (33). Our studies showed that DO expression in mouse DCs resulted in a profound decrease in self-pMHC at the cell surface (31, 32). We considered the possibility that DO expression in mouse DCs might sufficiently alter MHCII presentation of islet antigens to protect mice from T1D. To test this, the CD11c-DO transgene was introduced into a spontaneous mouse model for T1D, the Non-Obese Diabetic (NOD) mouse. CD11c-DO transgenic NOD mice (NOD.DO) displayed an altered set of self-pMHCII and were completely protected from T1D (32). Protection was not likely due to a change in central tolerance as NOD.DO mice had pathogenic T cells. Regulatory T cell function was unaltered and, therefore, not involved in protection. Perhaps most remarkably, Rag-1 deficient NOD.DO mice did not develop diabetes after receiving fully activated, pathogenic CD4 T cells from NOD mice. Thus, protection from T1D was due to DO inhibition of H2-M, which resulted in the inefficient presentation of islet-derived self-peptides by DCs. The identification what islet peptides are modulated by DO expression remains to be determined. Overall, these studies show that DO expression can shape the overall self-pMHCII repertoire in an immunologically relevant manner that promotes T cell tolerance and prevents autoimmunity.

If the physiological function of DO/H2-O in the immune system is to ensure that a broad, low-level of self-pMHC complexes are presented as a mechanism to promote self tolerance, then H2-O deficient mice should develop autoimmune disease. Consistent with this, a recent study showed that older H2-O deficient mice developed high IgG2a titers specific for dsDNA, ssDNA, and histones, autoantibodies specificities often found in lupus (29). H2-O deficient mice did not develop to full blown autoimmune disease though, and did not develop renal disease, which is often observed in lupus. Importantly, transfer studies showed that autoantibody development required H2-O deficient CD4 T cells. These data support that H2-O deficiency results in the selection of a self-reactive CD4 T cell repertoire that provides B cell help. However, the direct impact of DO/H2-O in modulating the self-pMHCII repertoire presented by thymic epithelial cells in the thymus, the cells that mediate T cell selection, has yet to be directly assessed by direct sequencing of MHCII-bound peptides from wild type and H2-O deficient mice. Collectively, the finding that loss of H2-O in mice results in the development of antinuclear antibodies together with our data showing that DO expression in mouse DCs prevents T1D support that DO/H2-O inhibition of H2-M alters self-pMHCII presentation to promote self tolerance and avoid autoimmunity.

## Conclusion

The presentation of disease relevant self-pMHCII (MHCII) molecules by APC is essential for the initiation of many autoimmune diseases. The repertoire of peptides bound to MHCII is regulated positively by DM and negatively by DO. DO squelching DM activity ensures that a diverse, low-level of self-pMHC are presented at the cell surface which promotes tolerance and protects from autoimmune disease development. Further experimentation is warranted to substantiate these ideas and to better understand the immunological consequences of DO inhibition of DM activity.

## Conflict of Interest Statement

The author declares that the research was conducted in the absence of any commercial or financial relationships that could be construed as a potential conflict of interest.

## References

[1] Mathis D Benoist C Back to central tolerance. Immunity (2004) 20:509–16 10.1016/S1074-7613(04)00111-6 15142520

[2] Karlsson L DM and DO shape the repertoire of peptide-MHC-class-II complexes. Curr Opin Immunol (2005) 17:65–70 10.1016/j.coi.2004.11.003 15653313

[3] Berger AC Roche PA MHC class II transport at a glance. J Cell Sci (2009) 122:1–4 10.1242/jcs.035089 19092054 PMC2714400

[4] Blum JS Wearsch PA Cresswell P Pathways of antigen processing. Annu Rev Immunol (2013) 31:443–73 10.1146/annurev-immunol-032712-095910 23298205 PMC4026165

[5] Riberdy JM Newcomb JR Surman MJ Barbosa JA Cresswell P HLA-DR molecules from an antigen-processing mutant cell line are associated with invariant chain peptides. Nature (1992) 360:474–7 10.1038/360474a0 1448172

[6] Ghosh P Amaya M Mellins E Wiley DC The structure of an intermediate in class II MHC maturation: CLIP bound to HLA-DR3. Nature (1995) 378:457–62 10.1038/378457a0 7477400

[7] Jensen PE Weber DA Thayer WP Chen X Dao CT HLA-DM and the MHC class II antigen presentation pathway. Immunol Res (1999) 20:195–205 10.1007/BF02790403 10741860

[8] Pos W Sethi DK Wucherpfennig KW Mechanisms of peptide repertoire selection by HLA-DM. Trends Immunol (2013) 34:495–501 10.1016/j.it.2013.06.002 23835076 PMC3796002

[9] Painter CA Stern LJ Conformational variation in structures of classical and non-classical MHCII proteins and functional implications. Immunol Rev (2012) 250:144–57 10.1111/imr.12003 23046127 PMC3471379

[10] Pos W Sethi DK Call MJ Schulze MS Anders AK Pyrdol J Crystal structure of the HLA-DM-HLA-DR1 complex defines mechanisms for rapid peptide selection. Cell (2012) 151:1557–68 10.1016/j.cell.2012.11.025 23260142 PMC3530167

[11] Alfonso C Karlsson L Nonclassical MHC class II molecules. Annu Rev Immunol (2000) 18:113–42 10.1146/annurev.immunol.18.1.113 10837054

[12] Liljedahl M Kuwana T Fung-Leung WP Jackson MR Peterson PA Karlsson L HLA-DO is a lysosomal resident which requires association with HLA-DM for efficient intracellular transport. EMBO J (1996) 15:4817–24 8890155 PMC452218

[13] Denzin LK Sant’Angelo DB Hammond C Surman MJ Cresswell P Negative regulation by HLA-DO of MHC class II-restricted antigen processing. Science (1997) 278:106–9 10.1126/science.278.5335.106 9311912

[14] van Ham SM Tjin EP Lillemeier BF Gruneberg U van Meijgaarden KE Pastoors L HLA-DO is a negative modulator of HLA-DM-mediated MHC class II peptide loading. Curr Biol (1997) 7:950–7 10.1016/S0960-9822(06)00414-3 9382849

[15] Denzin LK Fallas JL Prendes M Yi W Right place, right time, right peptide: DO keeps DM focused. Immunol Rev (2005) 207:279–92 10.1111/j.0105-2896.2005.00302.x 16181343

[16] Chen X Laur O Kambayashi T Li S Bray RA Weber DA Regulated expression of human histocompatibility leukocyte antigen (HLA)-DO during antigen-dependent and antigen-independent phases of B cell development. J Exp Med (2002) 195:1053–62 10.1084/jem.20012066 11956296 PMC2193689

[17] Glazier KS Hake SB Tobin HM Chadburn A Schattner EJ Denzin LK Germinal center B cells regulate their capability to present antigen by modulation of HLA-DO. J Exp Med (2002) 195:1063–9 10.1084/jem.20012059 11956297 PMC2193692

[18] Chen X Reed-Loisel LM Karlsson L Jensen PE H2-O expression in primary dendritic cells. J Immunol (2006) 176:3548–56 16517723 10.4049/jimmunol.176.6.3548

[19] Hornell TM Burster T Jahnsen FL Pashine A Ochoa MT Harding JJ Human dendritic cell expression of HLA-DO is subset specific and regulated by maturation. J Immunol (2006) 176:3536–47 16517722 10.4049/jimmunol.176.6.3536

[20] Fallas JL Yi W Draghi NA O’Rourke HM Denzin LK Expression patterns of H2-O in mouse B cells and dendritic cells correlate with cell function. J Immunol (2007) 178:1488–97 17237397 10.4049/jimmunol.178.3.1488

[21] Porter GW Yi W Denzin LK TLR agonists downregulate H2-O in CD8alpha- dendritic cells. J Immunol (2011) 187:4151–60 10.4049/jimmunol.1003137 21918198 PMC3186832

[22] Guce AI Mortimer SE Yoon T Painter CA Jiang W Mellins ED HLA-DO acts as a substrate mimic to inhibit HLA-DM by a competitive mechanism. Nat Struct Mol Biol (2012) 20:90–8 10.1038/nsmb.2460 23222639 PMC3537886

[23] Yoon T Macmillan H Mortimer SE Jiang W Rinderknecht CH Stern LJ Mapping the HLA-DO/HLA-DM complex by FRET and mutagenesis. Proc Natl Acad Sci U S A (2012) 109:11276–81 10.1073/pnas.1113966109 22733780 PMC3396517

[24] Kropshofer H Vogt AB Thery C Armandola EA Li BC Moldenhauer G A role for HLA-DO as a co-chaperone of HLA-DM in peptide loading of MHC class II molecules. EMBO J (1998) 17:2971–81 10.1093/emboj/17.11.2971 9606180 PMC1170637

[25] Liljedahl M Winqvist O Surh CD Wong P Ngo K Teyton L Altered antigen presentation in mice lacking H2-O. Immunity (1998) 8:233–43 10.1016/S1074-7613(00)80475-6 9492004

[26] Perraudeau M Taylor PR Stauss HJ Lindstedt R Bygrave AE Pappin DJ Altered major histocompatibility complex class II peptide loading in H2-O-deficient mice. Eur J Immunol (2000) 30:2871–80 10.1002/1521-4141(200010)30:10<2871::AID-IMMU2871>3.0.CO;2-B 11069069

[27] Draghi NA Denzin LK H2-O, a MHC class II-like protein, sets a threshold for B-cell entry into germinal centers. Proc Natl Acad Sci U S A (2010) 107:16607–12 10.1073/pnas.1004664107 20807742 PMC2944729

[28] McHeyzer-Williams LJ Pelletier N Mark L Fazilleau N McHeyzer-Williams MG Follicular helper T cells as cognate regulators of B cell immunity. Curr Opin Immunol (2009) 21:266–73 10.1016/j.coi.2009.05.010 19502021 PMC2731669

[29] Gu Y Jensen PE Chen X Immunodeficiency and autoimmunity in H2-O-deficient mice. J Immunol (2012) 190:126–37 10.4049/jimmunol.1200993 23209323

[30] Gondre-Lewis TA Moquin AE Drake JR Prolonged antigen persistence within nonterminal late endocytic compartments of antigen-specific B lymphocytes. J Immunol (2001) 166:6657–64 11359820 10.4049/jimmunol.166.11.6657

[31] Fallas JL Tobin HM Lou O Guo D Sant’Angelo DB Denzin LK Ectopic expression of HLA-DO in mouse dendritic cells diminishes MHC class II antigen presentation. J Immunol (2004) 173:1549–60 15265882 10.4049/jimmunol.173.3.1549

[32] Yi W Seth NP Martillotti T Wucherpfennig KW Sant’Angelo DB Denzin LK Targeted regulation of self-peptide presentation prevents type I diabetes in mice without disrupting general immunocompetence. J Clin Invest (2010) 120:1324–36 10.1172/JCI40220 20200448 PMC2846047

[33] Anderson MS Bluestone JA The NOD mouse: a model of immune dysregulation. Annu Rev Immunol (2005) 23:447–85 10.1146/annurev.immunol.23.021704.115643 15771578

